# Identification of a new bovine viral diarrhea virus subtype in the Republic of Korea

**DOI:** 10.1186/s12917-018-1555-4

**Published:** 2018-08-07

**Authors:** Du-Gyeong Han, Ji-Hyung Ryu, Jinho Park, Kyoung-Seong Choi

**Affiliations:** 1 Department of Animal Science and Biotechnology, College of Ecology and Environmental Science, Kyungpook National University, Sangju, 37224 Republic of Korea; 2 College of Veterinary Medicine, Chonbuk National University, Iksan, 54596 Republic of Korea

**Keywords:** Bovine viral diarrhea virus, 5′-untranslated region, BVDV-1o

## Abstract

**Background:**

Bovine viral diarrhea virus (BVDV) is prevalent in Korean indigenous cattle, leading to substantial economic losses. This study was conducted to investigate the occurrence of BVDV. In 2016, a total of 143 blood samples were collected from asymptomatic Korean indigenous calves younger than 3-months of age from six different farms in the Republic of Korea (ROK).

**Results:**

Eighty-seven calves (60.8%, 87/143) were tested positive for BVDV as evaluated by RT-PCR analysis. Phylogenetic analysis based on the 5′-untranslated region was used to classify these cases into three subtypes: BVDV-1b, BVDV-1o, and BVDV-2a. These results showed that BVDV-1b was the predominant subtype, while 2 samples clustered with BVDV-2a. Interestingly, one sample formed a separate group as a potentially new subtype, BVDV-1o. To our knowledge, this is the first report of BVDV-1o infection in Korean native calves. The BVDV-1o subtype identified in this study was closely related to cattle isolates obtained from Japan, indicating that this subtype is a new introduction to the ROK.

**Conclusions:**

This study provides useful information for carrying out epidemiological surveys of BVDV in the ROK and developing a vaccine for future use in the ROK, particularly for the first detection of BVDV-1o in Korean indigenous calves. Further studies are required to investigate the prevalence and pathogenicity of this BVDV-1o subtype.

## Background

Bovine viral diarrhea virus (BVDV) causes significant economic losses worldwide in the cattle industry through decreased productive performance and immunosuppression of herds [1, 2]. BVDV belongs to the genus *Pestivirus* along with classical swine fever virus and border disease virus in the family *Flaviviridae*. BVDV infects not only cattle, but also pigs, goats, sheep, and wild ruminants. BVDV includes two species, BVDV-1 and BVDV-2. Additionally, HoBi-like pestivirus has been proposed as a third new species, BVDV-3. Based on the 5′-untranslated region (UTR), BVDV-1 can be further divided into 21 subtypes (1a−1u) [3], BVDV-2 into four subtypes (2a−2d) [4, 5], and BVDV-3 into two genotypes of Brazilian and Thai origin [6]. Infection with BVDV is characterized by gastroenteritis, respiratory diseases and reproductive problems including abortion, congenital abnormalities, and the development of persistently infected (PI) calves that gain immunotolerance to BVDV through vertical transmission of the virus during early gestation [1, 7, 8].

To date, seven BVDV subtypes (1a, 1b, 1c, 1d, 1 m, 1n, and 2a) have been identified in the Republic of Korea (ROK) [9, 10]. Of these, BVDV-1b and BVDV-2a are predominant and widespread in cattle in the ROK [10–12]. The genetic diversity of BVDV must be considered when designing and constructing effective vaccination strategies against the virus. Additionally, a vaccine must accurately reflect the antigenic subtypes present in the country of use [13]. In the present study, we report a new subtype recently identified in the ROK. The results provide useful information for vaccine development.

## Methods

### Sample collection

**Fig. 1:**
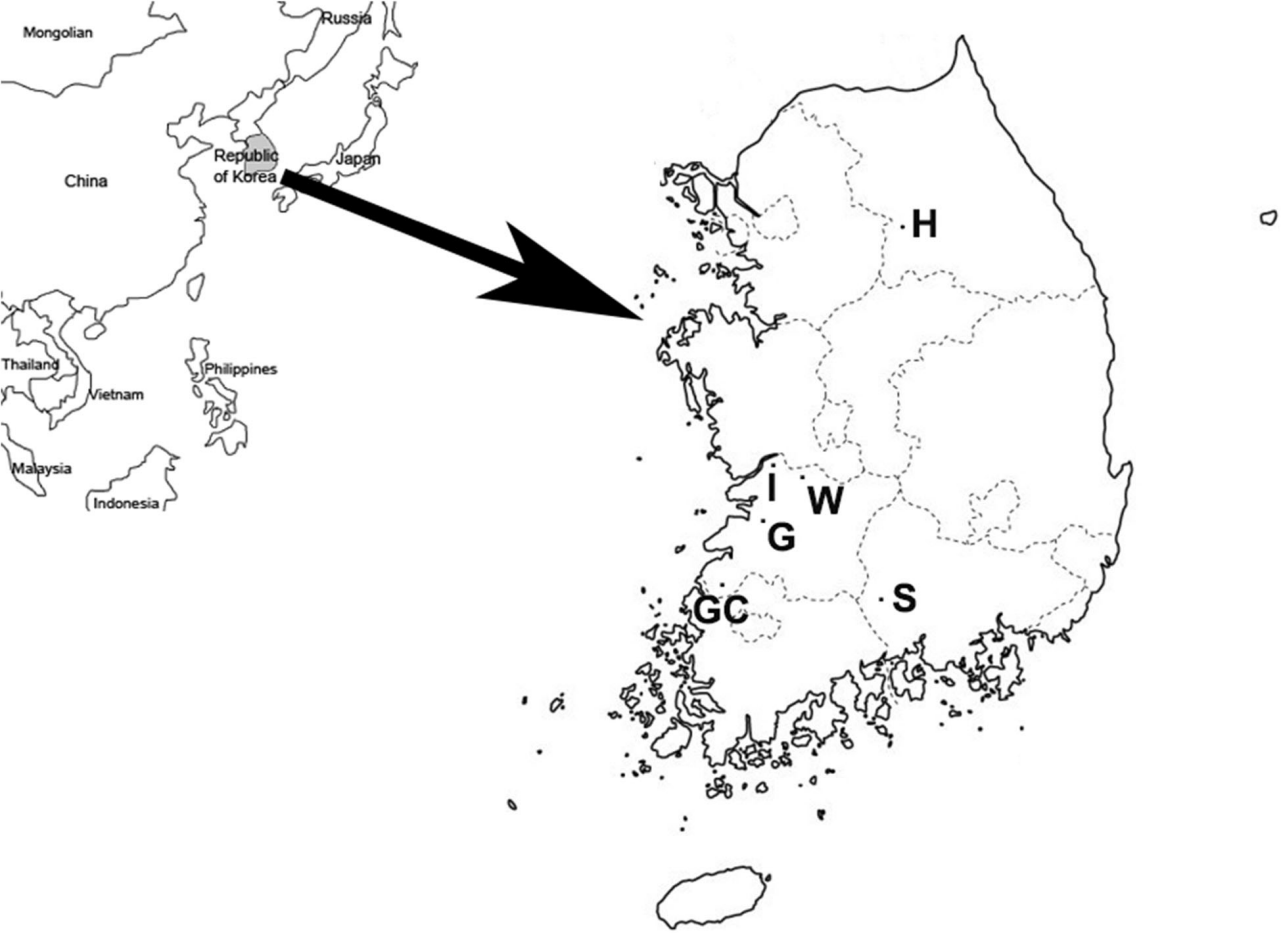
Map of the Republic of Korea. Dots indicate the regions where blood samples were collected: G, Gimje; GC, Gochang: I, Iksan; W, Wanju; H, Hoengseong; and S, Sancheong

Blood samples were collected from 143 asymptomatic Korean indigenous calves under 3 months of age from six different farms (Gimje, Gochang, Iksan, Sancheong, Wanju, and Hoenseong) in the ROK (Fig. 1). Five milliliters of blood was collected from the jugular veins of each calf in EDTA-supplemented tubes. The samples were delivered to the lab immediately after blood collection. These calves were the offspring of cows that had not previously been vaccinated against BVDV. The collected samples were stored at −80°C until analysis.

### RNA extraction, RT-PCR, and sequencing

Total RNA was extracted from 200 μL of blood using RNAiso Plus Reagent (Takara Bio, Shiga, Japan) according to the manufacturer’s instructions. RNA was eluted in 20 μL of RNase-free water and stored at −80°C. The 5′-UTR and N-terminal protease (N^pro^) regions were used to detect BVDV as previously described [4, 11]. Reverse transcription-polymerase chain reaction (RT-PCR) was performed to amplify BVDV using DiaStar™ One-Step RT-PCR Smart Mix (Solgent, Daejeon, Korea). Briefly, RT-PCR was carried out at 50°C for 30 min, followed by 94°C for 5 min, and then 30 cycles at 94°C for 30 s, 55°C for 30 s, and 72°C for 1 min, with a final extension step at 72°C for 10 min. Distilled water was used as a negative control in each PCR run. The predicted sizes of the amplified PCR products were 288 bp and 425 bp for the 5′-UTR and for N^pro^, respectively. The amplicons were subjected to 1.5% agarose gel electrophoresis and visualized following ethidium bromide staining. The PCR products were purified using an AccuPrep® PCR Purification Kit (Bioneer, Daejeon, Korea) and cloned into the pGEM^®^-T Easy vector (Promega, Madison, WI, USA), which was directly sequenced (Macrogen, Inc., Seoul, Korea).

### Phylogenetic analysis

For homology analysis of BVDV genes, the obtained sequence data were analyzed using the Basic Local Alignment Search Tool of the National Center for Biotechnology Information database. Homologous sequences were analyzed using Chromas software (version 2.33, http://www.technelysium.com.au/chromas.html) and aligned using ClustalX (version 1.8). Construction of the phylogenetic trees based on the 5′-UTR and N^pro^ were performed using the maximum-likelihood method with a Kimura 2-parameter [14] substitution model using MEGA7 software [15]. To construct each phylogenetic tree, additional sequences were obtained from GenBank (http://www.ncbi.nlm.nih.gov). The 5′-UTR of the gene sequences obtained in this study was assigned the following accession numbers: MH355922−MH355950, MH396616, and MH396618 for BVDV-1b, and MH396617 and MH396619 for BVDV-2a. The 5′-UTR and N^pro^ for the gene sequence of BVDV-1o obtained in this study were assigned the accession numbers MF449421 and MG030482, respectively.

### Statistical analysis

Statistical analysis was performed to assess the association between BVDV infection and the variables: colostrum intake and grazing. Binary univariate logistic regression models were constructed using the generalized linear mixed model in SPSS 24.0 software package (SPSS, Inc., Chicago, IL, USA), with the farm as a random effect. Odds ratios (ORs) with 95% confidence intervals (CIs) were calculated to assess the likelihood of association. A value of *P* < 0.05 was considered significant.

## Results

From the analysis of 143 blood samples, BVDV was detected in 87 calves (60.8%) from five of the six farms sampled (Table 1). Data for colostrum intake and growth type from the farms in the regions where fecal samples were collected are presented in Table 1. The Korean native calves examined in this study were born to cows that were not vaccinated against BVDV. There was no significant difference between colostrum intake and BVDV infection (*P* = 0.09). In calves that had not been fed colostrum (Heongseong and Iksan), BVDV infection was reduced by grazing (Iksan). In the Iksan and Sancheong regions where animals are grazed, colostrum-fed calves (Sancheong) showed an even higher rate of BVDV infection than in Iksan, where calves had not been fed colostrum (Table 1). Our results show that grazing decreased the OR for the incidence of BVDV by 5-fold compared to housing calves (OR = 0.2; 95% CI: 0.1−0.4; *P* = 0.001). BVDV infection showed the highest prevalence (64.7%) in 1−21-day-old animals and the prevalence of BVDV infection gradually reduced with age, but remained high (Table 2).

**Table 1:** Information in the regions where fecal samples were collected

Farm	No. of samples	BVDV positive	Subtype	Colostrum intake	Growth type
Gimje	20	14 (70.0%)	1b	Yes	Housing
Gochang	20	16 (80.0%)	1b	Yes	Housing
Wanju	62	42 (67.7%)	1b	Yes	Housing
Sancheong	14	7 (50.0%)	1b	Yes	Grazing
Hoengseong	20	8 (40.0%)	1b, 1o, 2a	No	Housing
Iksan	7	0 (0.0%)	−	No	Grazing
Total	143	87 (60.8%)

**Table 2:** Prevalence of BVDV infection according to age group of calves

Age group (days)	No. of examined calves	No. of BVDV positive
1−21	51	33(64.7%)
22−42	54	33 (61.1%)
43−84	38	21 (55.3%)
Total	143	87 (60.8%)

**Fig. 2:**
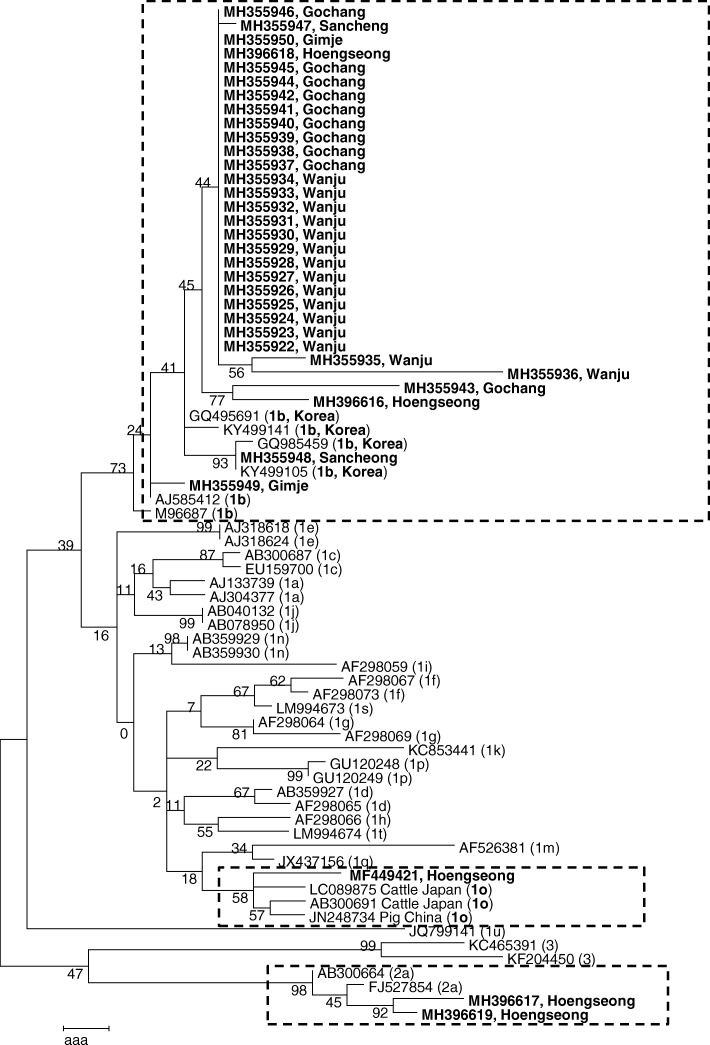
Phylogenetic tree based on partial nucleotide sequences of the 5′-UTR of reference BVDV strains/isolates and Korean isolates identified in this study was constructed in MEGA7 using the maximum-likelihood (ML) method. The robustness of the tree was evaluated by bootstrapping 1000 replicates by ML. The Korean isolates identified in this study are indicated in bold

**Fig. 3:**
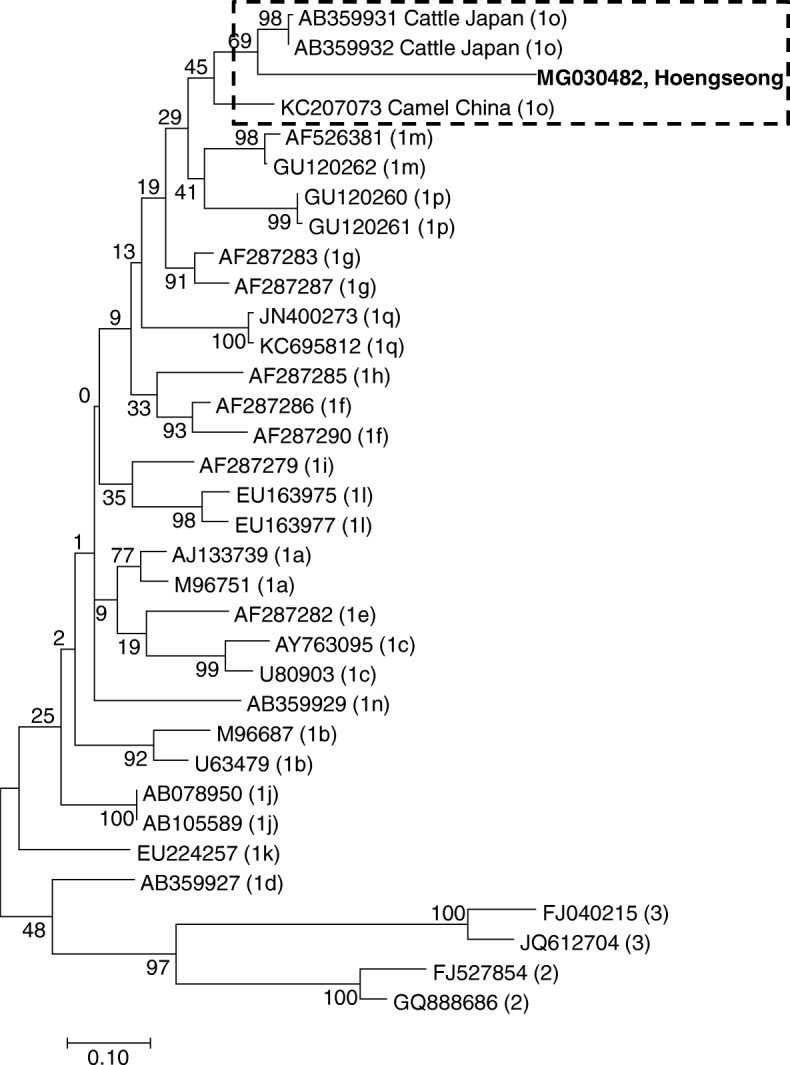
Phylogenetic tree of the N^pro^ region from Korean isolate identified in this study was constructed in MEGA7 by the maximum-likelihood (ML) method. The robustness of the tree was evaluated by bootstrapping 1000 replicates by ML. The Korean isolate identified in this study is indicated in bold

To investigate the genetic relationship between the BVDV isolates identified in this study, we sequenced all amplicons. Of these, 34 good sequences for 5′-UTR and one sequence for N^pro^ were obtained and were included in the phylogenetic tree. Thirty-four isolates identified from Gimje, Gochang, Sancheong, Wanju, and Hoengseong were classified into 3 subtypes: BVDV-1b (31 isolates), BVDV-1o (1 isolate, new subtype), and BVDV-2a (2 isolates) based on sequence analysis of 5′-UTR (Fig. 2). Particularly, in case of Hoengseong farm, three subtypes were detected simultaneously. Phylogenetic analysis revealed genetic differences in samples derived from the same farm. Two isolates of BVDV-2a were detected in this study and were similar to those that had previously been isolated in the ROK. The BVDV-1o found in Hoengseong farm was closely related to that identified in Japanese cattle (LC089875) (Fig. 2). Another phylogenetic tree based on the N^pro^ region was constructed to confirm the subtyping of BVDV-1o. As shown in Fig. 3, the Hoengseong isolate formed the same branch as BVDV-1o as determined by the sequence of 5′-UTR. Additionally, higher similarity was observed for most isolate relationships, except for the camel isolate. Homology values between the Hoengseong isolate identified in this study and BVDV-1o reference strains LC089875 and AB359931 were 95 and 88% for the 5′-UTR sequence and N^pro^ sequence, respectively. This is the first report of BVDV-1o infection in calves in the ROK. The results demonstrated that BVDV-1b was the most frequently detected subtype and genetic variations were evident among BVDV-1b circulating in the ROK (Fig. 2).

## Discussion

In this study, we investigated the occurrence of BVDV in Korean indigenous calves. Our findings showed that the infection rate of BVDV was high, indicating the difficulty in controlling the spread of BVDV in the field. Unexpectedly, the highest rate of BVDV infection was observed in 1−21-day-old calves. Most BVDV infections in calves less than 30-days-old are likely PI. In this study, we could not examine whether these calves were PI or acutely infected because the farmers objected to a second collection of blood from the calves. Previous reports from several countries have highlighted the clinical significance of BVDV infections, particularly of PI [16, 17]. Because PI animals remain unidentified, they can contribute to the spread of BVDV to other members of the herd. Measures to prevent BVDV circulation in herds should focus on removing the PI animals. Alterations in the immune system due to BVDV infection result in an increased susceptibility to other enteric diseases in PI calves [18, 19]. Because PI animals are important for the ongoing transmission of BVDV in a population, our findings highlight the need for rapid diagnosis and elimination of PI animals.

Our findings showed that BVDV infection was not associated with colostrum intake (*P* = 0.09). However, BVDV infection significantly decreased when animals were allowed to graze in a pasture (*P* = 0.001) rather than being confined to housing. In this study, BVDV infection was not detected in the Iksan region, where animals grazed. Interestingly, on the Sancheong farm, where animals also grazed, the prevalence of BVDV was relatively higher than that on the Iksan farm (Table 1). This difference between the two farms may be related to colostrum intake. The prevalence of BVDV was high in colostrum-fed calves, supporting the possibility that cows that served as sources of colostrum were infected with BVDV or PI. Moreover, the high prevalence of BVDV in calves can be explained by the failure to vaccinate. In the ROK, vaccination of pregnant dams against BVDV is not implemented on most farms. This study highlights the importance of BVDV vaccination. Consequently, an outbreak of BVDV may commonly be associated not only with grazing but also with other factors, including cattle management systems, herd sizes, and vaccination. Further studies are necessary to determine the effects of these variables in reducing the occurrence of BVDV.

Although the number of samples was limited, our results revealed that three subtypes (BVDV-1b, BVDV-1o, and BVDV-2a) of BVDV were detected in Korean native cattle. Of these, BVDV-1b was the predominant subtype identified. Our data support those of previous studies [10, 11]. However, the BVDV isolates obtained in this study showed more genetic variation compared to those in other Korean BVDV-1b strains/isolates reported previously in the ROK. Additionally, two isolates were classified as BVDV-2a based on phylogenetic analysis. The BVDV-2a outbreak is known to be associated with severe acute infections in the ROK [12], but the isolates were detected in calves without any clinical symptoms. Recent studies demonstrated that BVDV-2c causes severe and often fatal illness in cattle in European countries [20, 21]. Thus, disease outbreaks appear to change over time. BVDV-1o was identified for the first time in the present study, and a total of eight subtypes of BVDV (1a, 1b, 1c, 1d, 1 m, 1n, 1o, and 2a) have now been found in the ROK, including BVDV-1o newly detected. This study provides useful information for constructing a vaccine for future use in the ROK, particularly for detecting the BVDV-1o subtype in Korean indigenous calves. BVDV1o was first isolated from a calf that developed a mucosal disease and from PI calves in Japan [22, 23], and has been detected in camels, goats, and pigs in China [3, 24, 25]. However, this isolate has not been detected anywhere other than Japan and China. Our results indicate that BVDV-1o is genetically heterogeneous but geographically restricted. At present, we cannot conclude how this virus spread. It is possible that trade between countries could be a possible pathway for the introduction of the new subtype into Korean cattle.

## Conclusions

Our results provide evidence of the predominance of BVDV-1b in Korean native calves. Additionally, we identified the BVDV-1o subtype, which had not been previously documented in the ROK. As the number of calves examined was limited, larger epidemiological surveys are needed to investigate the prevalence of the BVDV-1o subtype as well as other subtypes. These results provide useful information for the development an effective vaccination for BVDV control.

## Abbreviations

5′-UTR: 5′- untranslated region
BVDV: Bovine viral diarrhea virus
ML: Maximum-likelihood
N^pro^: N-terminal protease
PI: Persistent infection
ROK: Republic of Korea
RT-PCR: Reverse transcription-polymerase chain reaction
